# Antitumor Activity of the Carboplatin-α-Cyclodextrin Adduct

**DOI:** 10.1155/MBD.1997.305

**Published:** 1997

**Authors:** Weiping Liu, Huizhou Xiong, Yikun Yang, Ling Li, Zhiqiang Shen, Zhihe Chen

**Affiliations:** 1 Institute of Precious Metals Kunming 650221 China; 2 Kunming Medical College Kunming 650031 China

## Abstract

Antitumor activity of the adduct between Carboplatin and α-cyclodextrin has been tested against Sarcoma 180 (S^180^) and Ehrlich ascites carcinoma (EAC) murine tumors. The preliminary toxicity has also been evaluated by histological examinations of the treated animals. The results show the adduct has less antitumor efficacy than and similar toxicity to Carboplatin.


### ANTITUMOR ACTIVITY OF THE CARBOPLATIN-α-CYCLODEXTRIN ADDUCT

Weiping Liu*[1], Huizhou Xiong[1], Yikun Yang[1], Ling Li[2], Zhiqiang Shen[2], and Zhihe Chen[2]

[1] Institute of Precious Metals, Kunming 650221
[2] Kunming Medical College, Kunming 650031,China

**Abstract**
Antitumor activity of the adduct between Carboplatin and α-cyclodextrin has been tested against Sarcoma 180 ($S_{180}$) and Ehrlich ascites carcinoma (EAC) murine tumors. The preliminary toxicity has also been evaluated by histological examinations of the treated animals. The results show the adduct has less antitumor efficacy than and similar toxicity to Carboplatin.

### 1. Introduction

Carboplatin,diammine [1,1-cyclobutanedicarboxylato]platinum(II), is a well known antitumor platinum drug. At present, it is provided in the form of a lyophilised powder which has to be reconstituted into an injectable solution form just before administration. Unfortunately the resulting solution is stable for only 8 hours at room temperature[1]. After this period, it is recommended that the solution should be discarded. Consequently the methods for improving the aqueous stability of Carboplatin merit consideration. It has been reported that Carboplatin can be included into α-cyclodextrin as a 1:1 adduct [2][3] and that the inclusion markly increases aqueous stability of Carboplatin [4]. Whether the inclusion affects its antitumor activity remained an open question. In order to further evaluate the possibility of using clinically this adduct, we have studied the influence of inclusion on the antitumor activity and preliminary toxicity of Carboplatin, and report our results here.

### 2. Experimental part

The adduct of Carboplatin into α-cyclodextrin was synthesized and purified according to a reported method [4]. Its molecular formula was determined by elemental analysis and MS-FAB to be $[(NH_3)_2Pt(C_6H_6O_4).(C_6H_{10}O_5).8H_2O]$ (experimental values: Pt 13.1 5%, C 34.07%, N 1.84%, H 5.95%, MS-FAB$^+$ m/e 1489 (M+1); calculated values: Pt 13.10%, C 33.90%, N 1.88%, H 5.92%, molecular weight 1488), the content of Carboplatin being 25% in the adduct. Female KM mice weighing 18-20 grams, and two murine tumor models $S_{180}$ and EAC were used in the experiments. The adduct and free Carboplatin were dissolved immediately before use in sterile 5% glucose. Mice bearing tumors were randomized into drug groups and control group. Each group had 15 mice. The control group was treated with the drug vehicle by the same route as drug groups. $S_{180}$ cells were transplanted to 45 mice by injection into the left axillary site. 2 days later,the drugs were administrated ip for 8 days and then tumors were dissected out and accurately weighed. The weights of drug and control groups were compared. Liver, spleen, kidney and lung of the mice were sumitted for histological examinations. Mice were transplanted ip with EAC cells, and 2 days later administrated iv with the drugs for 6 days. Within a 60 day experiment,the survival were recorded and activity of the drugs was assessed as the increase in life span. The tissues of the mice surviving for 28-30 days were also taken out for histological examinations.

### 3. Results and disscussion

The antitumor activity of the adduct and of Carboplatin in the corresponding two murine tumor models is listed in Table 1 from which it can be seen that, although the adduct exhibits considerable antitumor efficacy, it is obvisiously less effective than Carboplatin at equivalent doses. This means that the inclusion of α-cyclodextrin reduces antitumor activity of Carboplatin. No differences among the adduct and Carboplatin in pathological changes were observed, so the toxicity of the adduct is probably comparable to that of Carboplatin. The reason for the decrease in antitumor efficacy of the adduct is not yet understood but may be related to the difficulty in cellular uptake due to the larger size of the adduct and/or to the dissociation equilibrium between included and free Carboplatin, Carboplatin being only partly released from the adduct. The adduct shows no superiority over Carboplatin in activity and toxicity except its aqueous stability. Therefore there remains doubt of possible clinical uses of the adduct.

Table 1. Antitumor activity of Adduct and Carboplatin against $S_{180}$ and EAC murine tumors

| Drug | Tumor | Schedule | Dose (mg/kg) | T/C% Tumor Weight | T/C% Life Span |
|---|---|---|---|---|---|
| Carboplatin | $S_{180}$ | ip daily x 8 | 30 | 70.7% | |
| | | | 15 | 58.5% | |
| Adduct | $S_{180}$ | ip daily x 8 | 120 (30)* | 53.7% | |
| | | - | 60 (15)* | 30.1% | |
| Carboplatin | EAC | iv daily x 6 | 15 | | 168% |
| Adduct | EAC | iv daily x 6 | 80 (20)* | | 153% |
| | | - | 60 (15)* | | 131 % |
| | | - | 40 (10)* | | 123% |

* Figures in brackets give the amount of Carboplatin in the adduct

**References**
1. Levlus P. Harol, *European Patent* 0,334,551, A1, 1987
2. D. R. Alston,T. H.Lilley, J. F.Stoddart, *J. Chem. Soc. Chem. Commun.*,1985,1600
3. D. R. Alston, A. M. Z.Slawin, J. F. Stoddart, *J. Chem. Soc. Chem. Commun.*,1985, 1602
4. Liu Weiping, Xiong Huizhou, Yany Yikun, *Chinese J . Pharm.*, 1995, **24**(Suppl.),45

**Received: August 19, 1997 - Accepted: September 11, 1997 - Received in revised camera-ready format: October 20, 1997**

